# Non-coding RNA interact to regulate neuronal development and function

**DOI:** 10.3389/fncel.2014.00047

**Published:** 2014-02-24

**Authors:** Bharat R. Iyengar, Ashwani Choudhary, Mayuresh A. Sarangdhar, K. V. Venkatesh, Chetan J. Gadgil, Beena Pillai

**Affiliations:** ^1^CSIR-National Chemical Laboratory, Chemical Engineering and Process Development DivisionPune, India; ^2^Department of Chemical Engineering, Indian Institute of Technology BombayMumbai, India; ^3^Functional Genomics, CSIR-Institute of Genomics and Integrative BiologyDelhi, India

**Keywords:** miRNA, piRNA, lncRNA, network-motifs, gene expression regulation

## Abstract

The human brain is one of the most complex biological systems, and the cognitive abilities have greatly expanded compared to invertebrates without much expansion in the number of protein coding genes. This suggests that gene regulation plays a very important role in the development and function of nervous system, by acting at multiple levels such as transcription and translation. In this article we discuss the regulatory roles of three classes of non-protein coding RNAs (ncRNAs)—microRNAs (miRNAs), piwi-interacting RNA (piRNAs) and long-non-coding RNA (lncRNA), in the process of neurogenesis and nervous function including control of synaptic plasticity and potential roles in neurodegenerative diseases. miRNAs are involved in diverse processes including neurogenesis where they channelize the cellular physiology toward neuronal differentiation. miRNAs can also indirectly influence neurogenesis by regulating the proliferation and self renewal of neural stem cells and are dysregulated in several neurodegenerative diseases. miRNAs are also known to regulate synaptic plasticity and are usually found to be co-expressed with their targets. The dynamics of gene regulation is thus dependent on the local architecture of the gene regulatory network (GRN) around the miRNA and its targets. piRNAs had been classically known to regulate transposons in the germ cells. However, piRNAs have been, recently, found to be expressed in the brain and possibly function by imparting epigenetic changes by DNA methylation. piRNAs are known to be maternally inherited and we assume that they may play a role in early development. We also explore the possible function of piRNAs in regulating the expansion of transposons in the brain. Brain is known to express several lncRNA but functional roles in brain development are attributed to a few lncRNA while functions of most of the them remain unknown. We review the roles of some known lncRNA and explore the other possible functions of lncRNAs including their interaction with miRNAs.

## Background

The complexity of the nervous system is evident at the organ-system level, cellular level and at the molecular level. The systems-level complexity of the neuronal system is due to the highly connected neuronal network wherein each neuron connects to many other neurons by establishing synapses. At the cellular level, the complexity of the neuronal system arises from the cellular heterogeneity of the vertebrate brain, carrying at least four distinctly different cell types-neurons including several biochemically diverse sub-classes, astrocytes, oligodendrocytes and microglia; that have specialized but interdependent functions. At the molecular level, the complexity arises from the ability to create functional diversity from the genome through mechanisms like alternative splicing and RNA editing that are more prevalent in the nervous system as compared to other tissues; which makes the brain have the most complex transcriptome (Ramsköld et al., 2009), compared to other organs. The fact that brain capacity and cognitive abilities have greatly expanded from invertebrates to humans with a much lesser increase in the number of protein coding genes, indicates that gene regulation plays a major role in the development and function of the nervous system.

It was a long held idea that proteins are the versatile catalysts of life processes and RNAs serve the role of relaying the genetic message for the protein output. However, some of the key cellular processes including the very process of protein synthesis, is controlled by RNAs and studies, over the years, have shown that the functional ability of RNAs is much more than what was previously assumed. The diverse class of non-protein coding RNAs (ncRNAs), including microRNAs (miRNA), piwi-interacting RNA (piRNA), long-non-coding RNA (lncRNA) etc, are mainly involved in regulation of gene expression and are thus integral parts of the gene regulatory network (GRN). The recent evidences demonstrating wide-spread transcription throughout the genome suggest that the non-coding RNA form a sizeable component of the transcriptome of a eukaryotic cell (Jacquier, 2009; Clark et al., 2011). Several recent reviews have comprehensively cataloged these regulatory ncRNAs and provide a wealth of emerging evidence regarding their biogenesis, interacting partners and ability to regulate key target genes (Liu and Paroo, 2010).

Here, after a brief introduction to these classes of non-coding RNAs, we focus on two critical aspects of non-coding RNA function in the development of the vertebrate nervous system: their expression pattern and the network architecture of their interactions with other genes. The regulatory nature of these interactions is derived as much from the inherent features of the network as from the identity and functions of the genes that form the network. This review therefore draws on the principles of systems biology to explore the network of non-coding RNA and target interactions in the context of the nervous system.

## Classes of non-coding RNAs

Currently, these non-coding RNAs are classified into functional groups, on the basis of the rather arbitrary criteria of size and a limited knowledge of their functional roles in the cell. The major classes of ncRNAs are lncRNA (Mercer et al., 2009), piRNA, endogeneous small interfering RNA (endo-siRNA) and miRNAs.

**Figure 1:**
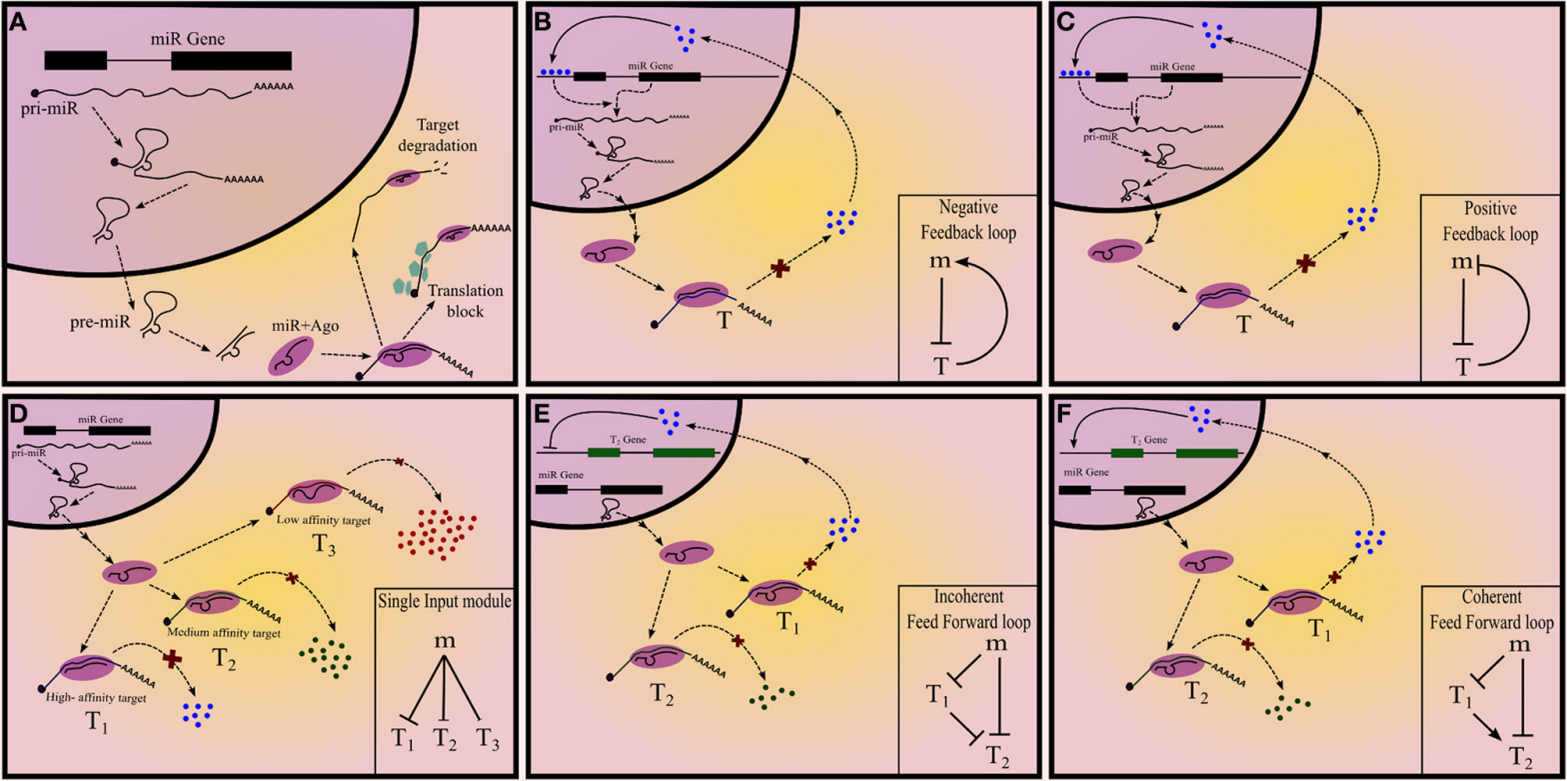
**Summary of different types of network motifs in miRNA mediated gene regulatory networks. (A)** Summary of miRNA biogenesis and mechanism of action. **(B)** Positive and **(C)** negative feedback loops, with a miRNA and a transcription factor. **(D)** Single input module with miRNA regulating three target nodes. The extent of regulation can differ between different targets. Feed-forward loops **(E)** Incoherent and **(F)** coherent feedforward loops with a miRNA regulating the target-T_2_.

miRNAs in their mature form are, on an average, 22 nt long and repress mRNAs which harbor miRNA “target sites” or miRNA recognition elements (MRE) in their 3′UTRs. These target sites are partially complementary to the miRNA and interact with the latter by Watson-Crick type base pairing. Functional miRNAs are bound to Argonaute (Ago) proteins and constitute a ribonucleoprotein complex called RNA Induced Silencing Complex (RISC), that is tethered to mRNAs by the miRNA-mRNA interaction. miRNAs arise from longer transcripts, called pri-miRNAs (primary miRNAs). Stem-loop like structures on pri-miRNA are identified by Drosha-DGCR8 (microprocessor) complex and are liberated from the long transcript by ribonucleolytic cleavage, to give rise to precursor miRNA (pre-miRNA). Pre-miRNA is exported to cytoplasm by Exportin-5 where it is again cleaved by Dicer to produce an imperfect RNA duplex. One or both the strands of the duplex is incorporated into Ago proteins to form a functional RISC (Figure 1A). Alternatively, miRNAs can also arise from introns of other transcripts as a result of splicing and subsequent cleavage by Dicer and these intronic regions are referred to as mirtrons (Filipowicz et al., 2008). The expression of miRNA can, therefore, be regulated at transcriptional or post-transcriptional levels (Winter et al., 2009). Even though the MREs are partially complementary to the miRNA, a 7-8mer region, called the seed, corresponding to 2nd–9th position of the miRNA, is essential for miRNA mediated regulation (Filipowicz et al., 2008). However, the partial complementarity allows the miRNA to target several mRNAs simultaneously (Chi et al., 2009; Helwak et al., 2013), forming a network motif called single input module (SIM) (Figure 1D). This may help in canalization of the gene regulation program (Hornstein and Shomron, 2006).

piRNAs are slightly longer than miRNA (24–32 nt long) and associate with Piwi family of proteins which include Piwi, Aubergine (Aub) and Ago3 in Drosophila and MIWI, MILI, and MIWI2 in mouse. Even though the binding partners of piRNAs are structurally similar to the miRNA-binding Ago proteins, the piRNA biosynthetic pathway is very different from that of miRNA. piRNAs arise from specific genomic loci known as piRNA clusters and check transposon expansion. Initially piRNAs were reported in the Drosophila and Mouse germline (Ishizu et al., 2012); subsequently they were also reported in somatic tissues like ovarian follicular cells of Drosophila and nervous system of mouse (Lee et al., 2011) and Aplysia (Rajasethupathy et al., 2012). Another class of piRNAs is predominantly expressed in mouse spermatids during pachytene stage of meiosis I. This class of piRNA is expressed in high abundance in these cells but its functions and putative targets, remain unknown. piRNA clusters store transposon derived sequences; transcripts arising from these clusters associate with Piwi-proteins (Piwi and Aub in Drosophila and MIWI and MILI in mouse) and are trimmed from the 3′ end to generate primary piRNAs. The 3′ end is 2′-O-methylated which makes the piRNA stable. Primary piRNA can undergo a cycle of amplification, called ping-pong cycle, to generate secondary piRNAs (Ishizu et al., 2012). Unlike Drosophila where cleavage is sufficient to repress transposons, piRNA-mediated transposon repression predominantly happens via DNA methylation in mouse (Aravin et al., 2008; Watanabe et al., 2011). The exact mechanism of this process is yet to be explored.

lncRNAs are a diverse set that are loosely defined as ncRNAs longer than 200 nt that lack apparent protein coding ability due to the absence of a relatively long uninterrupted open reading frame. Therefore, unlike miRNAs or piRNAs, the lncRNAs differ greatly from each other with respect to size, interacting partners and mode of action. Most lncRNA regulate gene expression by affecting chromatin dynamics or providing scaffold/tether to regulatory proteins (Mercer et al., 2009). lncRNAs typically have the same structural features as mRNAs such as the 5′ cap, polyadenylated 3′ tail and undergo splicing to give rise to the final product. They are localized both to the nucleus and cytoplasm, but the signals that drive their localization are not known. The genomic loci of lncRNA can provide clues to their regulatory targets as it is observed that genes that are regulated by an lncRNA are usually located very proximal to it on the genome. Unlike miRNAs, they are not highly conserved and their primary sequence has not provided much information about their function. Recently, it has been shown that several lncRNAs maybe spliced at their 5′ and 3′ ends to form circular RNAs (Memczak et al., 2013). However the functional importance of circularization, presumably for increased stability, has not been established.

miRNAs can interact with lncRNA (Jalali et al., 2013) and circular RNAs (Hansen et al., 2013; Memczak et al., 2013) besides their mRNA targets suggesting that in the cell, these ncRNAs may exist as a network of mutually regulating entities. They can possibly serve as sinks that sequester each other temporarily, or even target each other for degradation by the formation of dsRNA.

There are several lines of evidence which suggest that some of these ncRNAs have functional roles in the brain. Regulation by miRNAs is well known in the brain where they regulate neurogenesis and synaptic plasticity. Moreover, mRNAs in brain have extended 3′UTRs, which suggests that the component of regulation by miRNA or other forms of post-transcriptional mechanisms is higher in the brain compared to other tissues (Ramsköld et al., 2009; Miura et al., 2013). Brain also expresses several lncRNAs (Mercer et al., 2008) and is the only non-germline associated somatic tissue presently known to express piRNAs (Lee et al., 2011; Rajasethupathy et al., 2012). In this article we discuss how these three classes of ncRNAs regulate development of nervous system and maintenance of its activities. We primarily emphasize on the role of these ncRNAs as a part of the GRN and how their connectivity determines the functional output. We shed light on the effects of miRNA mediated regulation when they are present in certain types of network motifs.

## Network motifs

In network terminology, each interacting entity is called a node and the interactions are called edges. A GRN is a directed network of gene products (including proteins and RNAs), in which certain genes control the expression of other genes. Since the interaction can either activate (positive) or repress (negative) the “target” gene, the edges of a GRN carry a sign along with direction. It is evident that for a given set of nodes several theoretically possible networks can be defined by taking all the combinations of edges from one gene to the other. However, real networks form a small subset of this large number of theoretically possible networks. Network motifs are the patterns of connections that are highly prevalent in real networks compared to what would be expected in a random network with the same set of nodes. This indicates that network motifs were selected over other patterns of connections because of the unique functions that the former can perform as a result of their structure. Network motifs form functional modules in a real network such as GRN: some common network motifs in the GRN include feedback loops (FBL) (autoregulation), feedforward loops (FFL), SIM, multiple input modules and dense overlapping regulons (Alon, 2007).

A gene “X” can regulate a target gene “Y” via multiple intermediates; the sign of the path from X to Y is the product of the signs of all the intermediate edges. If there is a path from a gene back to itself then the resultant network is called a FBL and based on the sign of this path there are two types of FBL: positive and negative, which perform distinct functions (Figures 1B,C).

In a FFL a node targets another node via two parallel branches. If the sign of both the branches are similar then the FFL is called coherent; otherwise incoherent. There are four types each of coherent and incoherent FFLs. Some types of FFL are more commonly observed than the others and are therefore well studied (Figures 1E,F). Like in the case of FBL, each type of FFL is associated with a unique function.

SIM is a network motif in which one node targets at least two nodes (Figure 1D). This is exemplified by one transcription factor or miRNA regulating many genes simultaneously. (Readers are encouraged to refer to Alon, 2007 for details on network motifs in GRN).

Since a gene is a part of a network, its regulation (or the regulation it exerts) cannot be studied in isolation. The study of the entire GRN would be cumbersome and intensive; however, because of the modular nature of the network motifs, they can be studied in isolation and their dynamic effect on the GRN can be predicted. Most of the network motifs discussed in this article are local; these in turn can be a part of a larger motif.

## miRNAs

miRNAs and transcription factors are the best studied components of the GRNs that underlie many developmental gene expression programs. Both of them can modulate the expression of multiple targets, alter cell fate and are often engaged in mutually reinforcing functions. However, miRNAs differ from transcription factors in many critical ways (Hobert, 2008). Firstly, almost all the known miRNAs are repressors while transcription factors are either repressors or activators and in some rare cases can act as both depending on the target and interacting partners. Secondly, miRNAs usually bring about downregulation of their targets by a post-transcriptional mechanism, by degrading the target RNA or blocking its translation. Transcription factor interaction with target DNA is largely mediated through structural elements while miRNA interaction with targets is largely governed by the rules of nucleic acid complementarity and are therefore more easily predicted. When a particular gene is targeted by a transcription factor there is usually a single or at most a few tandemly repeated sites present at that locus. However, a typical miRNA-target interaction is characterized by miRNA molecules that have to bind to several messenger RNA molecules. Lastly, transcription factors are usually not consumed in the transcription factor-DNA interactions and may indeed engage in multiple rounds of regulation. The fate of the miRNA engaged in miRNA-target complexes is not understood with similar clarity.

A general principle derived from empirical studies is that, usually, the lifetime of a response is inversely correlated with the response speed. Cell signaling responses, which usually rely on post-translational modifications or conformational changes of proteins, are fast but transient whereas transcriptional or epigenetic responses are long-lived but have a slow response. Post-transcriptional mechanisms, such as miRNA-mediated regulation, fall between these two extremes; faster than transcriptional regulation and relatively stable compared to cell signaling responses. The slow response time of transcriptional repression also results because of the continued presence of stable messenger RNAs since the already existing mRNAs continue to produce proteins. Indeed RNA degradation signals have evolved to ensure rapid turn-over of certain messenger RNAs but they do not allow regulation of the turn-over. miRNAs, can accelerate the response by rapidly clearing these mRNAs along with minimizing the effect of leaky transcription (Hornstein and Shomron, 2006). Therefore miRNA mediated regulation may be preferred over transcriptional regulation in certain situations while, in other situations it may by used along with the latter as a reinforcement.

**Figure 2:**
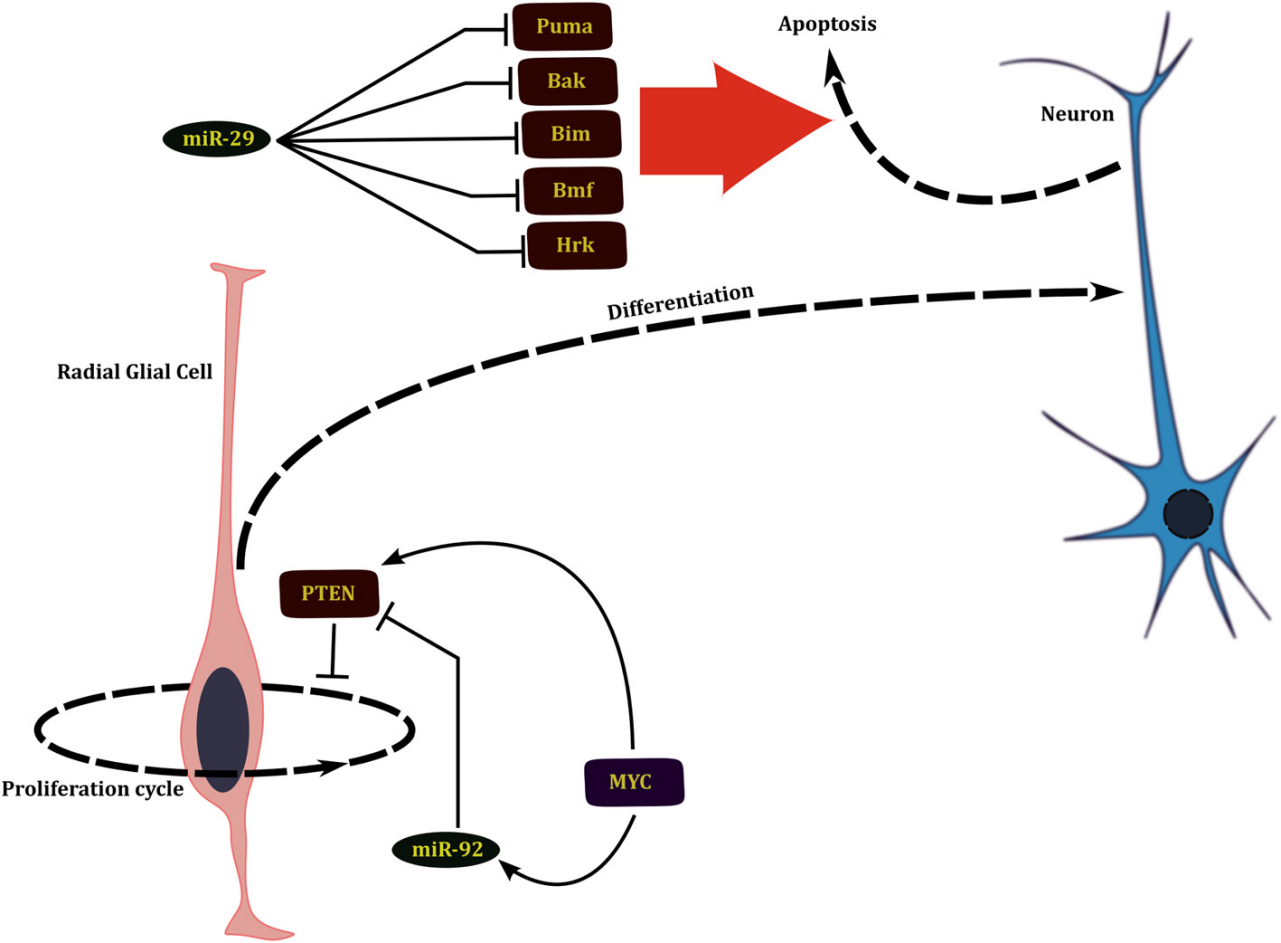
**miRNAs affect the development of nervous system by regulating proliferation of neural progenitors and apoptosis of young neurons**.

The miRNAs that play an important role in regulation of the neuronal system act at three levels that in turn correspond to three different developmental time scales. During the earliest stages of development of the nervous system, the numbers of neural stem cells are determined through a wave of neural stem cell proliferation followed by a phase of massive apoptosis (Buss and Oppenheim, 2004). Several miRNAs regulate the development of nervous system by either promoting cell division by repressing pro-apoptotic genes or later promote apoptosis by shutting down pro-survival signals during this early phase of development. miR-29b targets multiple BH3 family of pro-apoptotic genes such as Bim, Bmf, Puma, Bak, and Hrk during early neural differentiation (Figure 2). miR-29b mediated repression of apoptosis in the surviving healthy neurons is essential for neural development (Kole et al., 2011).

**Figure 3:**
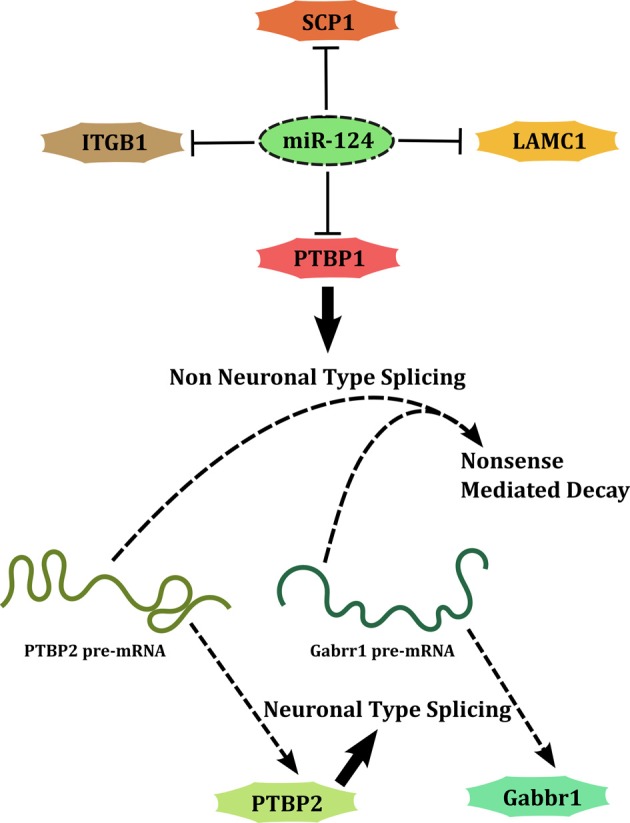
**miR-124 promotes neuronal differentiation by acting through a SIM.** PTBP1 is downstream of miR-124 and is a part of another SIM.

A second set of miRNAs regulate the development of nervous system by either promoting differentiation (developmental function) or allowing the initial expansion of neural progenitors (maintenance function). miR-124 and miR-9 are classical examples of miRNAs associated with developmental functions. miR-124 targets several genes including Poly-Pyrimidine Tract Binding Protein-1 (PTBP1) (Makeyev et al., 2007), Small C-terminal domain Phosphatase 1 (SCP1/CTDSP1) (Visvanathan et al., 2007), Laminin-γ (LAMC1) and Integrin-β1 (ITGB1) (Cao et al., 2007), to promote neuronal differentiation (Figure 3). This type of interaction (also the previously discussed case of miR-29b) forms a network motif called SIM in which a single factor acts on multiple downstream genes. This network architecture allows coordinated regulation of a set of targets that are perhaps simultaneously required to be cleared. Each of these targets in turn may target several genes thus amplifying the scope of regulation. An interesting case is that of PTBP1, a splicing factor, whose downregulation by miR-124 causes a global change in alternative splicing patterns, leading to expression of several neuronal transcript variants. In transcription factor based networks, it has been shown that as the concentration of the transcription factor increases over time following an activating signal, the targets maybe switched on, one after the other in the order of their affinity for the factor (Kalir et al., 2001). Although such an evidence of temporal regulation does not exist for miRNA encoded SIMs, the understanding of the properties of a SIM allows a general extrapolation. When the expression of an miRNA is knocked down partially, the targets with the lowest affinity are likely to be relieved of repression while high affinity targets may continue to be repressed. Attempts to target miRNAs for therapeutic applications have to consider this aspect of miRNA mediated targeting.

**Figure 4:**
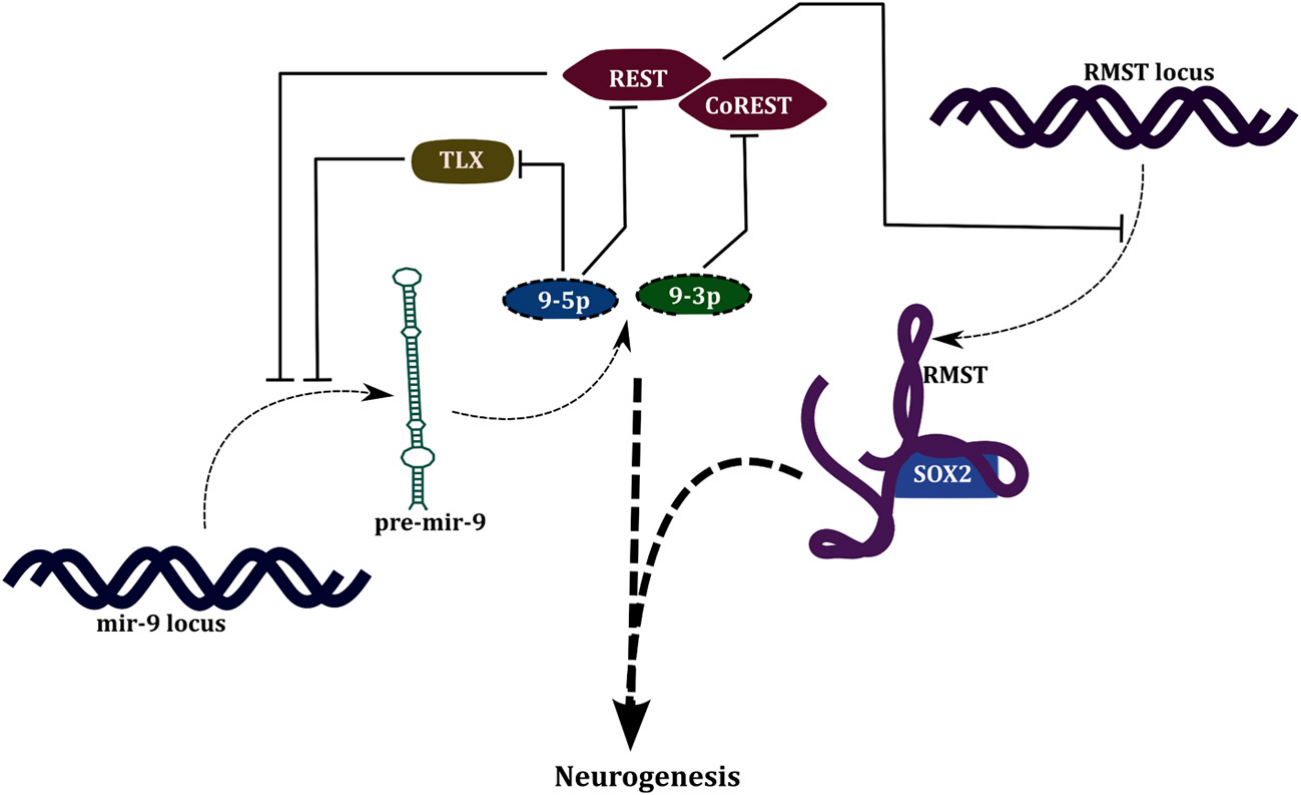
**Regulation of neurogenesis by miR-9 and lncRNA- RMST**.

The products of the miR-9 precursor—miR-9-3p and miR-9-5p—target the transcription factors REST and CoREST, respectively (Packer et al., 2008). In the proliferating progenitors, REST-CoREST, transcriptionally repress all the miR-9 genes, miR-9-1/2/3, and other neuronal genes. This mutual repression between miR-9 and REST-CoREST gives rise to a positive FBL (PFBL). miR-9 is also involved in a PFBL with TLX (Figure 4); a factor that regulates proliferation of neural progenitors (Zhao et al., 2009). It can be observed that developmental functions of miRNAs are associated with network motifs like SIM and PFBL. Multiple targets of a miRNA in a SIM allows simultaneous action on several genes thereby canalizing the cell toward differentiation. PFBLs give rise to bistability; existence of two stable steady states, i.e., differentiated and stem cell state. Examples of PFBL mediated switching behavior is also evident in case of transcriptional regulation; the most famous example would be the switching between lytic and lysogenic phenotypes in λ-phage.

Neural development is also indirectly affected by regulation of self-renewal and expansion of neural progenitors. Phosphatidylinsositide 3-kinase (PI3K)/Akt pathway is involved in growth and self renewal of stem cells whose dysregulation is implicated in several cancers. This pathway is also involved in regulation of neurogenesis; brains of *Akt3* knockout mice are greatly reduced in size (Easton et al., 2005). PTEN, which is a negative regulator of this pathway, is in turn regulated by the miR-17–92 cluster of miRNAs and loss of these miRNAs leads to suppression of neural stem cell expansion (Bian et al., 2013). Network analysis by El Baroudi et al. (2011) reveals that both the miR-17–2 cluster and PTEN are positively regulated by MYC, constituting a type 1 incoherent FFL (1I-FFL) (Figure 2). One of the functions that miRNAs perform as a part of 1I-FFL is to buffer transcriptional noise (Osella et al., 2011).

miRNA are also involved in maintenance of neuronal function by regulating synaptic plasticity. Since neurons are highly polarized cells with the nucleus quite distant from the dendritic spines, a local regulatory mechanism is required near the synapses to control protein synthesis at these regions. In other words, a transcriptional regulation in response to synaptic signals would be delayed and therefore miRNA mediated regulation is of great importance in neurons. miR-134 (Schratt et al., 2006) and miR-132 (Wayman et al., 2008; Mellios et al., 2011) are known to regulate synaptic plasticity and the morphology of dendritic spines. miR-134 is also shown to localize at the dendritic spines and repress LIMK1 (Schratt et al., 2006). It is to be noticed that under these conditions, the miRNA mediated regulation doesn't cause transcript degradation but rather, causes a translational repression. RNA binding proteins such as Dnd1 (Kedde et al., 2007) and HuR (Kundu et al., 2012) are reported to reverse miRNA mediated translational repression in germline and liver tissue. Banerjee et al. (2009) have reported that MOV10, a component of RISC, is rapidly degraded via NMDA-receptor mediated signaling, in dendritic spines. This relieves certain mRNAs, including LIMK1 and LYPA1, from miRNA mediated regulation. miRNAs have also been shown to specifically localize in the axons (Sasaki et al., 2013; Hancock et al., 2014); however, the list of axonally enriched miRNAs reported by these two studies are non-overlapping. Hancock et al. have found that miR-132 promotes axonal extension in mouse dorsal root ganglionic (DRG) neurons, by targeting Rasa1. In other studies it has been shown that certain miRNAs like miR-9 (Dajas-Bailador et al., 2012) and miR-138 (Liu et al., 2013) inhibit axonal extension by targeting Map1b and SIRT1, respectively. miRNAs are also implicated in regulation of axon regeneration, post-injury. Injury to sciatic nerve leads to upregulation of miR-21 and miR-431 in the DRG. Also, it was shown that miR-21 and miR-431 promote neurite outgrowth in cultured DRG neurons by targeting Spry2 and Kremen1, respectively (Strickland et al., 2011; Wu and Murashov, 2013a). Taken together, these facts indicate that miRNAs can perform contrasting roles in axonal regulation. Kaplan et al. (2013) and Wu and Murashov (2013b) have extensively reviewed this aspect of miRNA function in the nervous system. Many miRNA are known to co-express with their targets in the neurons suggesting that they might be controlled by a common regulator (Tsang et al., 2007). The fact that miRNAs and their targets are spatiotemporally co-expressed suggests that, in neurons, miRNAs are preferred over transcriptional mechanisms for dynamic gene regulation.

## piRNA

piRNAs are a relatively new class of small non-coding RNAs originally discovered in mouse germline tissues. piRNAs are known to suppress transposable elements in the germline tissues, thereby protecting the germline DNA from deleterious mutations; dysregulation of piRNA pathway results in defects in germ cell proliferation and hence resulting in the loss of fertility. Although most studies on piRNA have been on its role in the germline, a few studies have reported their presence and function in somatic cells (Malone et al., 2009; Lee et al., 2011; Rajasethupathy et al., 2012).

The first evidence of piRNA in nervous system was reported by Lee et al. in which piRNAs in the mouse hippocampus were detected by deep sequencing of small RNA libraries and applying stringent criteria to filter other small RNA sequences including miRNAs (Lee et al., 2011). Some candidate sequences were also found to be associated with MIWI by real-time PCR. Further they showed the presence of the abundant piRNA complexes in the dendritic spines and the knockdown of piRNAs resulted in reduced spine density in the axons.

In another report it was shown that approximately 300 genomic regions encode for piRNAs in the neurons of the Aplysia (Rajasethupathy et al., 2012). It was shown that certain piRNAs are induced by serotonin (5-HT) signaling. Subsequently, using Piwi knockdown studies, it was found that piRNA pathway leads to methylation of CREB2, thereby reducing its expression and promoting memory formation. Even though Rajasethupathy et al. argue that the previous report of piRNA in the brain by Lee et al. is a misclassification and may have arisen because of RNA impurities, a careful analysis from multiple model systems is required to fully comprehend the role of piRNAs in the nervous system.

Despite these reports, the presence of piRNA in the brain is not fully justified. A possible clue about the functions of piRNA in the brain comes from the discovery of the L1 retrotransposons in the brain. These elements have been shown to be regulated during neuronal differentiation and hypothesized to give rise to neuronal heterogeneity and somatic mosacism in brain (Muotri et al., 2005; Coufal et al., 2009). It has been already shown that L1 retrotransposons are regulated by piRNAs in the germline tissues. The presence of both piRNAs and L1 retrotransposons in the brain suggests that the former may regulate the latter in the brain as well.

Recently, it has been shown that the there is extensive transposon expression in the αβ neurons of mushroom bodies in the Drosophila brain, compared to the adjacent neurons (Perrat et al., 2013). In contrast Ago3 and Aub show a low expression in the αβ neurons compared to the rest of the brain. Moreover, mutants of different piRNA pathway components, i.e., Aub, Ago3, and Armi showed elevated levels of transposon expression in the brain. Considering all these observations it can be proposed, though not conclusively stated, that piRNAs are present in the brain for controlling transposons and repeat derived RNAs, and thereby regulating somatic heterogeneity.

## lncRNA

lncRNAs have been reported in the brain and known to be associated with specific regions (Mercer et al., 2008). Many of these are found to be specifically expressed during the development of the brain and neural cell differentiation (Ng et al., 2012).

Although it is difficult to classify lncRNAs based on their structural or mechanistic features, lncRNAs are associated with specific roles that they play in the development and functioning of the nervous system therefore allowing a functional classification. There are two functional groups of lncRNAs associated with the development of nervous system—lncRNAs that promote either the self-renewal of neural stem cells or neural differentiation. Another group of lncRNAs are involved in the maintenance of the functioning of nervous system, such as regulation of synaptic activity.

In the previously mentioned study by Ng et al., it was found that three lncRNAs, designated as lncRNA_ES1, lncRNA_ES2, and lncRNA_ES3, are specifically associated with pluripotent stem cells (Ng et al., 2012) lncRNA_ES1 and lncRNA_ES3 had binding sites for the pluripotency associated transcription factors OCT4 and NANOG, and just NANOG respectively; it was further confirmed that knockdown of these proteins reduced the levels of these lncRNAs. The role of these lncRNAs in maintenance of pluripotency was confirmed when their knockdown resulted in reduced percentage of pluripotent cells. These lncRNA_ES1/2 were found to be associated with the PRC2 component SUZ12, suggesting that they indirectly regulate pro-differentiation genes by repressing their transcription.

In the same study four lncRNAs- RMST, lncRNA_N1, lncRNA_N2, and lncRNA_N3, were identified to be associated with neuronal differentiation and their knockdown resulted in reduction in number of neurons in culture along with a reduced expression of neuronal markers and increased expression of glial markers. Out of these, lncRNA_N2 was found to harbor neurogenesis associated miRNAs- miR-125b and let-7 in its intronic regions. In a subsequent study by the same group it was shown that RMST is transcriptionally repressed by REST (Figure 4). Also, it was shown that RMST associates with SOX2 and tethers it to promoters of neurogenetic genes like DLX1, NEUROG2 and ASCL1, thereby inducing their expression (Ng et al., 2013). DLX1 locus also encodes an antisense-lncRNA, DLX1AS which is expressed during neurogenesis in the subventricular zone of hippocampus, and positively regulates the expression of DLX1 and DLX2 (Ramos et al., 2013).

lncRNAs that are involved in maintenance activities regulate the general functioning of neurons and control processes such as synaptic signaling. BC1 which is a cytoplasmic lncRNA, localizes to dendrites and is involved in regulating the post-synaptic signaling, by repressing metabotropic glutamate receptor signaling (mGluR) induced local protein synthesis. BC1 represses translation initiation by interacting with eIF4A and poly-A binding protein (PABP), thereby disallowing the recruitment of the small ribosomal subunit to the mRNA (Wang et al., 2005). Loss of BC1 results in hyperexcitation of the neurons (Zhong et al., 2009) and is implicated in the mouse models of epilepsy (Gitaí et al., 2011). One of the genes induced by mGluR is Fragile-X mental retardation protein (FMRP), which is significantly upregulated in BC1 knockouts (Zhong et al., 2009). However, FMRP is also involved in controlling hyperexcitability of neurons by regulating protein synthesis and it has been shown that double knockout of FMRP and BC1 results in a more severe epileptic phenotype (Zhong et al., 2010) This suggests that FMRP supplements BC1 and may rescue any imbalance caused by fluctuations in BC1 activity.

BACE1 is a membrane protease which is implicated in Alzheimer's Disease by promoting cleavage of Amyloid Precursor Protein (APP) to form Amyloid-Beta 1-42 (Abeta 1-42) peptide. BACE1-Anti Sense (BACE1AS) RNA is partially complementary to BACE1 coding region (CDS) and renders stability to the mRNA, thereby leading to upregulation of Abeta 1-42. BACE1AS is in turn upregulated because of the cellular stress due to Abeta 1-42, thereby further increasing Abeta 1-42 via BACE1. This results in a PFBL (Faghihi et al., 2008), however, in this study the authors have called it a feedforward regulation. It had been subsequently shown that BACE1AS stabilizes BACE1 mRNA by masking a non-canonical target site for miR-485-5p in the CDS of BACE1 mRNA (Faghihi et al., 2010).

In another contrasting case of antisense lncRNA mediated regulation, BDNF-AS represses Brain Derived Neurotrophic Factor (BDNF) and results in restriction of neurite growth. Also, this regulation occurs at the level of transcription where BDNF-AS helps in recruiting EZH2, which is a part of Polycomb Repressive Complex 2 (PRC2) and marks histones with repressive lysine methylation- H3k4me3 (Modarresi et al., 2012).

There are many other lncRNAs implicated in the development and function of nervous system but the targets and mechanisms are unknown for a majority of them; Table 1 summarizes these different lncRNAs. In many cases lncRNAs emerge from the same genomic locus as their targets; this targeting is based on simple base pairing can happen at the level of either DNA or RNA. Some lncRNAs such as RMST act on several genes by tethering transcription factors to their promoters or serving as a scaffold for the assembly of RNA-binding proteins.

**Table 1:** **Summary of lncRNAs involved in the development of the nervous system**.

**lncRNA**	**Biological function**	**Mechanism**	**References**
Gomafu	Neurogenesis, oligodendrocyte lineage specification	Binds to QKI and SRSF1 and regulates splicing	Tsuiji et al., 2011; Qureshi et al., 2010
Anti-NOS2A	Neuronal differentiation	Represses NOS2A	Korneev et al., 2008
MALAT-1	Synaptogenesis	Splicing regulation	Bernard et al., 2010
EVF-2	Differentiation of GABAergic neurons	Activates Dlx-5/6	Bond et al., 2009
TUG1	Retinal differentiation	–	Young et al., 2005
NEAT1	Neuronal/oligodendrocyte differentiation	Paraspeckle integrity	Mercer et al., 2010
Sox8OT	Oligodendrocyte lineage commitment	–	Mercer et al., 2010
Nkx2.2AS	Oligodendrocyte differentiation	–	Tochitani and Hayashizaki, 2008
HAR1F	Neural development	Reelin upregulation	Pollard et al., 2006
HOTAIRM	Neuronal differentiation	–	Lin et al., 2011
SIX3OS	Retinal differentiation	Recruiting Ezh2	Rapicavoli et al., 2011
POU3F2	Neural stem cell proliferation	–	Ramos et al., 2013

## Concluding remarks

Recent studies have revealed the importance of RNA mediated gene regulation in diverse biological processes. There are some advantages of having ncRNAs as gene expression regulators compared to an exclusively protein-regulated GRN. Since there is no step of translation, the cost as well the time required for making an ncRNA would be less compared to that of a protein. ncRNAs can target genes based on simple base pairing interactions; therefore evolution of such a regulator has a higher likelihood.

miRNA based regulation has a special importance in the highly polarized neurons as they control local translation at the dendrites thereby preventing the delay that can arise in transcriptional responses because of RNA synthesis and transport to distant cellular regions. Even though the exact importance of piRNAs in the brain is not understood it is highly likely that they regulate retrotransposons. Unlike miRNAs and piRNAs that are mostly repressors, lncRNAs can either activate or repress a gene. Since the dsDNA has limited ability to adopt distinctive structures, the DNA binding specificity of transcription factors and other DNA binding proteins relies on a limited repertoire of DNA binding domains. Long RNAs on the other hand are structured and proteins can bind to specific structures more effectively. The sequence information in the RNA can provide additional specificity to the binding. Thus lncRNAs may efficiently bridge the interactions between protein and DNA. lncRNAs can also mark large genomic regions for epigenetic regulation as in the case of XIST. It has also been argued that it is the act of transcription and not the product per-se, that causes the regulation (Berretta and Morillon, 2009).

Although these regulatory RNAs act via different mechanisms, usually their roles are convergent with that of the protein based transcriptional regulation to ensure an efficient and foolproof control of gene expression. As evident in many studies, blocking the activity of these regulatory RNAs affects the gene regulation to different extents. Therefore it is undeniable that ncRNAs supplement the gene regulation by proteins and are not merely redundant pathways. A systems-level analysis of ncRNAs is essential to understand their precise roles and the ability to confer robustness to the GRN.

### Conflict of interest statement

The authors declare that the research was conducted in the absence of any commercial or financial relationships that could be construed as a potential conflict of interest.
